# Mass-spectrometry data for *Rhizoctonia solani* proteins produced during infection of wheat and vegetative growth

**DOI:** 10.1016/j.dib.2016.05.042

**Published:** 2016-05-27

**Authors:** Jonathan P. Anderson, James K. Hane, Thomas Stoll, Nicholas Pain, Marcus L. Hastie, Parwinder Kaur, Christine Hoogland, Jeffrey J. Gorman, Karam B. Singh

**Affiliations:** a CSIRO Agriculture, Floreat, Western Australia; b The University of Western Australia Institute of Agriculture, Crawley, Western Australia; c Protein Discovery Centre, QIMR Berghofer Medical Research Institute, Herston, QLD, Australia

**Keywords:** Fungal pathogenesis, Wheat, *Rhizoctonia solani*, Basidiomycete

## Abstract

*Rhizoctonia solani* is an important root infecting pathogen of a range of food staples worldwide including wheat, rice, maize, soybean, potato, legumes and others. Conventional resistance breeding strategies are hindered by the absence of tractable genetic resistance in any crop host. Understanding the biology and pathogenicity mechanisms of this fungus is important for addressing these disease issues, however, little is known about how *R. solani* causes disease. The data described in this article is derived from applying mass spectrometry based proteomics to identify soluble, membrane-bound and culture filtrate proteins produced under wheat infection and vegetative growth conditions. Comparisons of the data for sample types in this set will be useful to identify metabolic pathway changes as the fungus switches from saprophytic to a pathogenic lifestyle or pathogenicity related proteins contributing to the ability to cause disease on wheat. The data set is deposited in the PRIDE archive under identifier PRIDE: PXD002806.

**Specifications Table**

**Table:** 

Subject area	Biology
More specific subject area	Plant Pathology
Type of data	Figure, table
How data was acquired	Mass spectrometer LTQ-Velos Orbitrap (Thermo Scientific) with search engine Mascot version 2.4.1 used to map spectra to the *R. solani* WAC10335 genome.
Data format	Raw
Experimental factors	Proteins extracted from fungal cultures undergoing vegetative growth or during infection of wheat.
Experimental features	Extracted soluble proteins from fungal hyphae, membrane-bound proteins from fungal hyphae and proteins collected from the culture filtrate were subjected to mass spectrometry. Spectra were mapped to the *R. solani* WAC10335 genome gene models and six frame translation of the genome.
Data source location	Brisbane / Perth, Australia
Data accessibility	Data are within this article and have been deposited to the ProteomeXchange Consortium via the PRIDE partner repository with the dataset identifier PRIDE: PXD002806

**Value of the data**

• Identification of metabolic changes as the fungus switches from a saprophytic lifestyle to a pathogenic lifestyle within the host may be inferred by comparing proteins under the vegetative and infection conditions.
• In-depth survey of proteins secreted from the fungal pathogen, *Rhizoctonia solani*, into the culture filtrate. These proteins are likely to come in direct contact with the plant host and thus may play important roles in infection/pathogenicity.
• Comparison could be made between proteins identified in vegetative fungal cultures and during infection of wheat to identify proteins related to infection or compared with a protein set from other fungal pathogens to identify conserved or unique infection strategies.

## 1 Data

**Fig. 1:**
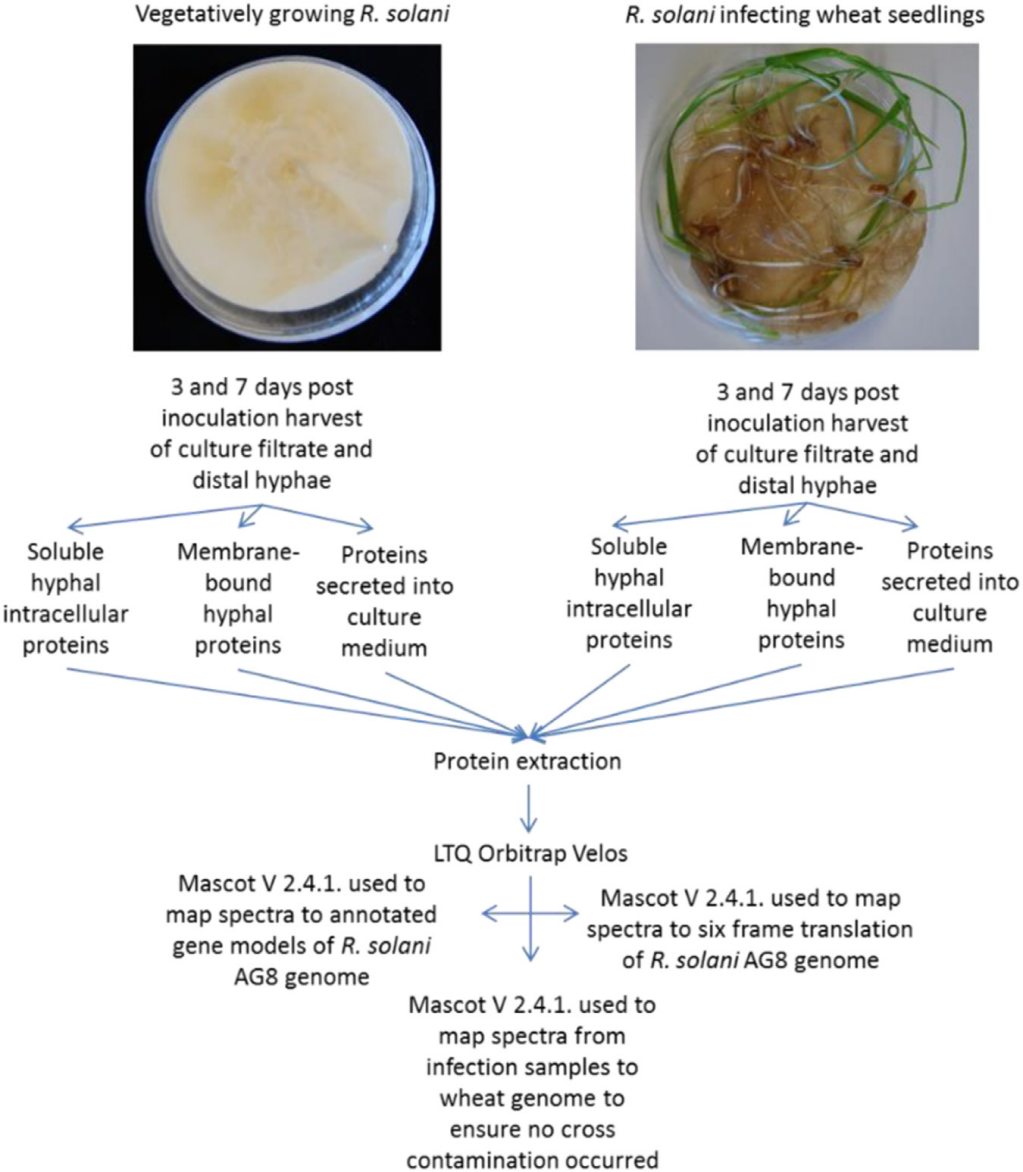
Experimental procedure for analysis of *R. solani* proteins produced under vegetative growth and wheat infection conditions.

This proteomics dataset comprises MS RAW files and identification files (mzIdentML and Scaffold files). MS/MS raw files were mapped to the *R. solani* AG8 WAC10335 gene models (GenBank assembly accession: GCA_000695385.1) and a six-frame translation of the genome using Mascot V2.4.1. Samples are obtained from either *R. solani* mycelium undergoing vegetative growth or *R. solani* infecting wheat seedlings at either early or late time points (Fig. 1).

## 2 Experimental design, materials and methods

### 2.1 Sample acquisition and generation of data

*Rhizoctonia solani* AG8 (WAC10335) [1] was allowed to grow at room temperature for 1 week on a PDA plate overlaid with a sterile nitrocellulose membrane. Surface sterilized wheat seeds were incubated at 24° C in the dark for 3 days on moist filter paper. Inoculations were conducted by adding the nitrocellulose membrane containing *R. solani* to the wheat seedlings and submerging in minimal medium [2]. The inoculated seedlings were incubated at 24° C for 3 days or 7 days prior to harvesting (Fig. 1). Vegetative fungal samples were obtained from cultures as above without the addition of wheat seedlings. Five replicates for each sample type (Table 1) were pooled for protein extraction and analysis. The membrane and attached mycelium was removed from the plates and mycelium peeled from the membrane, blotted dry and frozen in liquid nitrogen. Proteins were extracted from the culture filtrate, the membrane fraction of mycelium or the soluble fraction of mycelium as per [2]. Briefly, soluble mycelium and culture filtrate proteins were isolated using 10% (w/v) trichloroacetic acid, 0.07% (v/v) 2-mercaptoethanol in acetone and washed with 0.07% (v/v) 2-mercaptoethanol in acetone. Extraction of membrane proteins utilized a Mem-PER Plus Membrane Protein Extraction Kit (Thermo Scientific) followed by a 2D-Clean up kit (GE Healthcare). All proteins were trypsin digested and prepared for LC-MS according to [2].

### 2.2 LC-MS and data analysis

Trypsin-digested samples were analyzed on a Shimadzu Prominence nano HPLC system coupled to an LTQ-Velos Orbitrap ETD mass spectrometer controlled using Xcalibur 2.2 software (Thermo Fisher Scientific) according to [2]. All MS/MS samples were analyzed using Mascot (Matrix Science, London, UK; version 2.4.1) and SequestHT (Thermo Fisher Scientific, San Jose, CA, USA; version 1.4.1.14) using the annotated *R. solani* AG8-1 genome [3] (13952 entries) and the 6-frame translation (1,729,543 entries) or the wheat genome (ftp.ensemblgenomes.org/pub/plants/release-25/fasta/triticum_aestivum/dna/) databases, supplemented in all cases with the contaminants database (247 entries, downloaded from maxquant.org on Aug 26, 2013). Mascot and SequestHT were searched using the criteria described in [2]. Scaffold (version 4.1.1, Proteome Software Inc., Portland, OR), Peptide Prophet algorithm [4] and Protein Prophet [5] were used to validate peptide and protein identifications according to [2]. Proteins that contained similar peptides and could not be differentiated based on MS/MS analysis alone were grouped to satisfy the principles of parsimony.

## Figures and Tables

**Table 1:** Sample names and treatments.

Sample name	Sample time point Days post inoculation (dpi)	Condition	Protein location
PDC6C	3 dpi	Infection	Culture filtrate
PDC5G	3 dpi	Infection	Membrane
PDC5C	3 dpi	Infection	Soluble mycelium
PDC5F	3 dpi	Vegetative growth	Membrane
PDC5A	3 dpi	Vegetative growth	Soluble mycelium
PDC5E	7 dpi	Infection	Culture filtrate
PDC6F	7 dpi	Infection	Membrane
PDC5D	7 dpi	Infection	Soluble mycelium
PDC6B	7 dpi	Vegetative growth	Culture filtrate
PDC6E	7 dpi	Vegetative growth	Membrane
PDC5B	7 dpi	Vegetative growth	Soluble mycelium
